# ﻿The first checklist of fungi known for Honduras: revealing taxonomic, geographical, and functional trends

**DOI:** 10.3897/mycokeys.126.169230

**Published:** 2025-12-05

**Authors:** Libelje Mortier, Ruben Vanhoomissen, Lee Davies, Paul M. Kirk, Jose G. Maciá-Vicente, Meike Piepenbring, Danny Haelewaters

**Affiliations:** 1 Biology Centre of the Czech Academy of Sciences, Institute of Entomology, 370 05 České Budějovice, Czech Republic; 2 Department of Botany, Faculty of Science, University of South Bohemia, 370 05 České Budějovice, Czech Republic; 3 Research Group Mycology, Department of Biology, Ghent University, 9000 Ghent, Belgium; 4 Royal Botanic Garden, Kew, Richmond TW9 3AE, Surrey, UK; 5 Department of Marine Sciences and Applied Biology, University of Alicante, Apto. 99, 03080 Alicante, Spain; 6 Department of Microbial Ecology, Netherlands Institute of Ecology (NIOO-KNAW), 6700 AB Wageningen, The Netherlands; 7 Mycology Research Group, Goethe University Frankfurt am Main, Biologicum, 60438 Frankfurt am Main, Germany; 8 Centro de Investigaciones Micólogicas (CIMi), Universidad Autónoma de Chiriquí, Facultad de Ciencias Naturales y Exactas, Apartado Postal 0427, David, Panama; 9 Department of Zoology, Faculty of Science, University of South Bohemia, 370 05 České Budějovice, Czech Republic

**Keywords:** Biodiversity, Central America, conservation, ecology, Mesoamerica, mycology, species list, species richness

## Abstract

Fungi play pivotal roles in ecosystem functioning and the provision of ecosystem services. Despite their ecological importance, fungal research remains limited, particularly in tropical regions. Many tropical countries, including Honduras, still lack a comprehensive fungal checklist. To address this gap, we compiled the first fungal checklist for Honduras by collating data from Index Fungorum, MyCoPortal, the Kew Data Portal, and published literature. The resulting dataset contains 1365 species across 4011 records, of which 96.6% are true fungi and 3.4% are fungus-like organisms. Among the true fungi, 69.8% belong to Basidiomycota and 29.4% to Ascomycota. A large percentage of the records refer to plant pathogens (42.4%), reflecting a relatively high number of phytopathological studies and a focus on fungal associations with plant species. Species accumulation curves indicate that all administrative divisions (departments) of Honduras remain understudied, as none have reached saturation. This checklist is a fundamental resource tool for fungal identification and conservation planning and contributes to a broader understanding of fungal biodiversity in the region.

## ﻿Introduction

Many global efforts to conserve and restore nature, including activities of the International Union for Conservation of Nature (IUCN) and the Intergovernmental Panel on Climate Change (IPCC), rely for their policy recommendations on available scientific knowledge. International agreements such as the Kunming–Montreal Global Biodiversity Framework (GBF) advocate for protecting all species from extinction, including hyper-diverse groups such as fungi (Fenu et al. 2015). However, relative to other kingdoms of life, fungi are often neglected due to a lack of data regarding fungal diversity, distribution, persistence, and ecological roles in various ecosystems as well as a general underestimation of their importance (Hortal et al. 2015; Martin et al. 2022). This lack of data can be attributed to (i) their generally cryptic nature, with fungal microorganisms occupying ecological niches in very specialized substrates (Rambold et al. 2013), (ii) the fact that many areas and niches that are often hotspots for fungal diversity remain underexplored (Aime and Brearley 2012), and (iii) the lack of funding for micro-organismal research projects (Guénard et al. 2025), among other reasons. As such, the number of IUCN Red List assessments of fungi is low in comparison to most other multicellular organisms. As of 13 January 2025, 857 fungal species were assessed by the IUCN, compared to 91,667 animal species and 73,558 plant species (IUCN 2025). The number of fungal assessments also pales in comparison to their predicted diversity, estimated at 2.5 million (Niskanen et al. 2023; Haelewaters et al. 2024).

Tropical environments tend to exhibit higher levels of species richness and endemism in comparison to temperate regions (Smith et al. 2013; Bandala et al. 2016; Del Olmo-Ruiz et al. 2017; Mikryukov et al. 2023). Studies in Central America (Bermúdez and Sánchez 2000; Piepenbring 2007; Piepenbring et al. 2012) suggest that numerous fungal species remain undiscovered in tropical regions and biodiversity hotspots (Hawksworth and Lücking 2017). However, the number of extended fungal specimens from tropical regions is low in comparison to those from temperate regions (Lendemer et al. 2020; Quandt and Haelewaters 2021; Stallman et al. 2024a). Due to the lack of reference specimens and expertise, identifying and describing fungi from the tropics remain challenging (Piepenbring et al. 2020). Many tropical countries have no fungal species lists and have received little-to-no mycological attention, due to a lack of funding and infrastructure, among other reasons (Cazabonne et al. 2022).

One such understudied country is Honduras, part of the Mesoamerican biodiversity hotspot (Myers et al. 2000), where mycological research has been scarce, even compared to other Central American countries. To illustrate, Del Olmo-Ruiz et al. (2017) found a bare 12 fungal records from neotropical cloud forests in Honduras, compared to 2289 from the same ecosystem in Mexico and 1540 in Costa Rica. This contrast underscores the potential for significant fungal discoveries in Honduras, if more research initiatives were undertaken, of which a fungal checklist would be a logical starting point.

Piepenbring et al. (2020) presented a list of reasons as to why compiling fungal records into a unified database, i.e., a checklist of fungal species, is vital. A fungal checklist: (1) reveals the present state of mycology in an area; (2) is indispensable to decide whether fungal records are new for the area; (3) provides older (perhaps locally forgotten) names that may be revived by new collections; (4) helps to identify under sampled taxonomic and ecological groups as well as poorly explored geographical areas, and thereby yields arguments to justify (funding for) research projects; (5) gives the ability to compare diversity among regions and countries; and (6) helps to identify fungi collected in the area by providing possible names of species and references to literature for identification. The importance of species checklists is recognized under the 1992 Rio Convention on Biological Diversity, reinforcing their role in biodiversity characterization and management. A fungal checklist is essential to advance fungal research in Honduras and to inform us on how to best handle challenges related to climate change and habitat loss.

The urgency for fungal research and conservation in Honduras is high, as many areas in the country, even national parks, are subjected to alarming rates of habitat destruction (Martin et al. 2021). Deforestation resulting from logging is rampant in the Olancho Department, while the clearing of land for agriculture is prevalent in the La Mosquitia region. On a country scale, deforestation in 2016–2018 spanned 369.1 km^2^, with an average annual loss of 200 km^2^/year over the last decade (Interactive Country Fiches 2024). The coverage of mangrove swamps has continuously and rapidly decreased from 662.6 km^2^ in 1996 to 591.9 km^2^ in 2016 (Interactive Country Fiches 2024). A growing recognition of the utilitarian value of cloud forest ecosystems in Honduras, e.g., providing drinking water and preventing floods (IPBES 2019), resulted in the formal protection of all forests above an elevation of 1800 m a.s.l. in 1987 following the Cloud Forest Act (Act 87-1987) (Martin et al. 2021). However, without concrete conservation measures, these areas represent so-called paper parks without law-enforcing infrastructure (Martin and Blackburn 2009). For example, at least 20% of the core zone of Jeannette Kawas National Park has been lost due to expanding palm cultivation (DiBio 2017).

The goal of this study is to compile and analyze fungal records for Honduras from the available literature. Specifically, this study aims to: (1) evaluate the current knowledge of fungal species diversity; (2) discuss this knowledge from systematic, geographic, and historical viewpoints; and (3) identify gaps that need to be filled by future research.

## ﻿Methods

### ﻿Study area

Honduras is located in Central America and is bordered by Guatemala to the west, El Salvador and the Pacific Ocean (via the Gulf of Fonseca) to the south, Nicaragua to the east, and the Caribbean Sea to the north. Honduras is administratively divided into 18 departments: Atlántida, Choluteca, Colón, Comayagua, Copán, Cortés, El Paraíso, Francisco Morazán, Gracias a Dios, Intibucá, Islas de la Bahía, La Paz, Lempira, Ocotepeque, Olancho, Santa Bárbara, Valle, and Yoro. The capital, Tegucigalpa, is situated in the Francisco Morazán Department, in the south-central region of the country.

The climate in Honduras is tropical in the coastal lowlands with annual temperatures averaging 26 °C to 29 °C and becomes more temperate in the highlands where annual temperatures average 16 °C to 24 °C (Climate Change Knowledge Portal 2021). The average annual precipitation is lowest in the mountainous interior of the country (800–2000 mm) and highest at the Caribbean coast (≥2,000 mm). The Pacific coast and interior highlands have a dry season from November to April and a wet season from May to October, while the Caribbean coast has rainfall throughout the year (Climate Change Knowledge Portal 2021).

Honduras is home to a wide variety of ecosystems: rain forests, montane cloud forests, mangroves, savannas, mountain ranges with pine and oak trees, and the Mesoamerican Barrier Reef System. Honduras covers 112,492 km^2^ of which about 63,011 km^2^ comprises forests (approximately 56% of the national territory) (Interactive Country Fiches 2024). Forest cover (56%) is distributed as follows: 28.0% moist broadleaf forest, 17.3% coniferous forest, 10.3% deciduous broadleaf forest, and 0.5% mangrove forest. The territory is mostly mountainous, with slopes steeper than 30% and the mean elevation is 684 m a.s.l., with the highest peak being Cerro Las Minas, reaching 2878 m a.s.l. The country harbors an exceptionally high biodiversity, e.g., almost 8000 known species of plants, 276 reptiles, 153 amphibians, 771 birds, and 220 species of mammals, due to its location in the tropics and topographical conditions with a wide variety of niches (Interactive Country Fiches 2024). Main threats to Honduran biodiversity are habitat destruction and deterioration, ecosystem fragmentation, species overexploitation, exotic invasive species, and pollution (DiBio 2017; Martin et al. 2021; Interactive Country Fiches 2024).

### ﻿Collation of records and curation of the dataset

To compile the checklist of fungi known for Honduras, we used the following sources: scientific literature and three databases, i.e., the Mycology Collections data Portal (MyCoPortal), the Kew Data Portal, and Index Fungorum. Publications were found by searching Web of Science for the following terms: “Honduras” + “fungi”. We also searched for publications referencing Honduras or Central America in Lindau and Sydow (1908). Fungal records from MyCoPortal were downloaded after searching all available collections for specimens from Honduras (Locality Criteria: Country “Honduras”). The Kew Data Portal (http://data.kew.org/) was searched for Honduran collections deposited at the Kew Fungarium and digitized up to that date (25 October 2022). Finally, a list of species described based on material from Honduras was obtained from Index Fungorum (2025).

Duplicates were detected based on the record number (a number assigned by a given collector to a specific specimen) and subsequently deleted. Author names were standardized manually (e.g., “P.C. Standlay” to “P.C. Standley”). Scientific names were checked manually for completeness, typos, and taxonomic accuracy. Index Fungorum (2025) was used as a reference for fungal names. Locality resolution was chosen to be at the level of department, as many records did not provide more detailed information. Classification data were extracted from the National Center for Biotechnology Information (NCBI) Taxonomy Database (https://www.ncbi.nlm.nih.gov/taxonomy) and included the taxid number and taxonomic categories from kingdom to species. When the automatic search did not result in all additional information, it was added manually following the classification from Index Fungorum (2025). Only records identified up to the level of genus or species were retained. The final checklist (see Suppl. material 1) contains information for records of fungi and fungus-like organisms in Honduras in an Excel file, including taxonomy, locality, and record contexts.

### ﻿Analyses

Analyses were performed in R version 4.3.3 (R Core Team 2024). A Krona plot to visualize the taxonomic composition of the dataset was built using the “KronaTools” package (Ondov et al. 2011). A summary of the ecological guilds, based on genus-level occurrences, was constructed and data about the particular guild of each record were extracted from the FungalTraits database (Põlme et al. 2020). Species accumulation curves for every department were made in R using the *rarecurve* function of the “vegan” package version 2.6.4 (Oksanen et al. 2022). To illustrate the species richness through space, the richness for each department was plotted onto the country map of Honduras using QGIS (QGIS Development Team 2025). The following packages were used for additional analyses: “ggplot2” version 3.5.1 (Wickham 2016), “ggthemes” version 5.1.0 (Arnold 2024), “reshape2” version 1.4.4 (Wickham 2007), “sf” version 1.0.16 (Pebesma 2018; Pebesma and Bivand 2023), “RColorBrewer” version 1.1.3 (Neuwirth 2022), “dplyr” version 1.1.4 (Wickham et al. 2023), and “tidyr” version 1.3.1 (Wickham et al. 2024). Species richness estimators, Bootstrap (Efron 1979; Colwell and Coddington 1994), Jackknife 1 (Burnham and Overton 1978), and Chao1 (Chao 1984) were tested using the “specpool” function of the “vegan” package version 2.6.4 (Oksanen et al. 2022). Raw data are available online at https://github.com/dannyhaelewaters/haelewaters-group/tree/main/Honduras_checklist_of_fungi_2025.

## ﻿Results

A total of 4011 records representing 1365 species are included in the checklist of fungi known for Honduras. Of those records, 3782 originate from MyCoPortal, 70 from Index Fungorum, 65 from Royal Botanical Gardens, Kew, and 94 from the literature (spanning 35 papers). All records are accompanied by source information, indicating the origin of collection (MyCoPortal, Index Fungorum, Kew Data Portal, or published literature). Many records also include the locality (at the department level), collector information, and the year of sampling. A smaller subset of records contains additional metadata, including a collector’s number, detailed locality, and identifier information.

**Figure 1.**
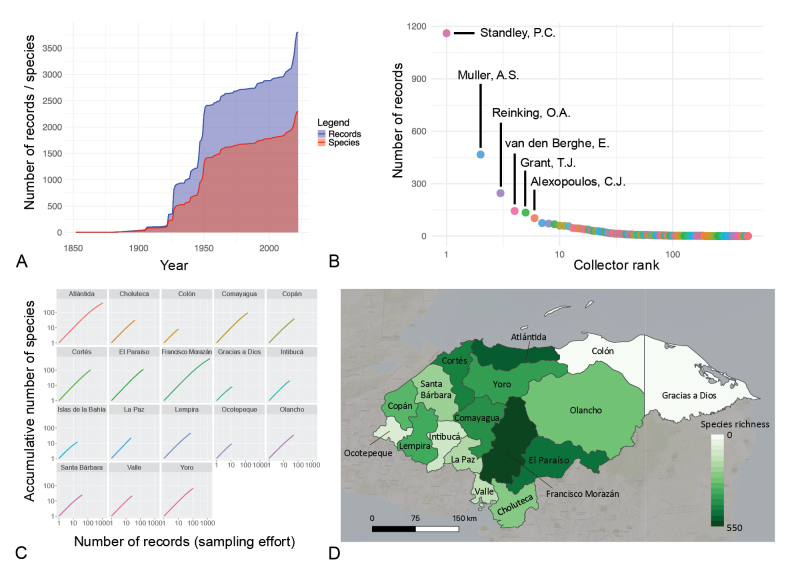
Analyses of species and records compiled in the checklist of fungi known for Honduras. A. The cumulative number of fungal records and species recorded through time; B. Rank abundance curve showing the number of records per collector. Names of the collectors with highest number of Honduran records in the checklist are indicated; **C.** Species accumulation curves for the 18 departments of Honduras. The accumulative number of species for each department is plotted in relation to the number of records; **D.** Map of Honduras and its departments indicating the recorded species richness per department.

A temporal increase is observed in both the number of fungal records (sampling effort) and fungal species detected in Honduras over the last 100 years (Fig. 1A). For 206 records the year is unknown; these were excluded from this analysis. Three steep increases in sampling effort can be detected as well as a similar but less pronounced pattern for observed species richness. The first records from Honduras date back as far as the second half of the 19^th^ century and were made by the botanist J.A. Hjalmarson. A small number of records were made between 1900 and 1920 by M.E. Peck and P. Willson. The period between 1923 and 1928 marks the first steep increase in sampling effort, mainly caused by the activities of three botanists: M.A. Carleton, P.C. Standley, and O.A. Reinking (Fig. 1A). The collectors identified above contributed a high number of records to the checklist; M.E. Peck, P. Willson, M.A. Carleton, P.C. Standley, and O.A. Reinking added 72, 47, 44, 1178, and 246 collections, respectively, placing them among the top 15 collectors (Fig. 1B). The second and largest increase in fungal sampling effort is observed around the 1940s–1950s, which coincides with the establishment of the Escuela Agrícola Panamericana (Zamorano University, est. 1942). Indeed, most records from this period are from the Francisco Morazán Department where Zamorano University is located. From then onwards, fungal sampling effort rises steadily, with a third steep increase initiating between 2012 and 2022. This can potentially be attributed to an increase in public interest in fungal biodiversity and to the inclusion of research-grade observations in the field uploaded to databases such as iNaturalist.

Species accumulation curves for the different departments of Honduras do not show saturation for any department, indicating that fungal documentation remains incomplete across all regions (Fig. 1C). As no extrapolation was carried out, some accumulation curves are shorter than others due to a low number of records in certain departments. In this study, a higher species richness is more likely a reflection of greater sampling effort rather than actual species diversity. The highest species richness in a department (Fig. 1D) is documented for Francisco Morazán (564 species, 1458 records), followed by Atlántida (399 species, 1034 records) and El Paraíso (112 species, 157 records). For Colón and Gracias a Dios, only 10 and 12 records (twice representing 8 species), respectively, have been reported to date. There is no locality information for 586 records; these records were excluded from this visualization. Species richness estimates for all departments are very tentative, showing high standard errors resulting from low sampling – both in the number of species and number of records (see Suppl. material 2).

**Figure 2.**
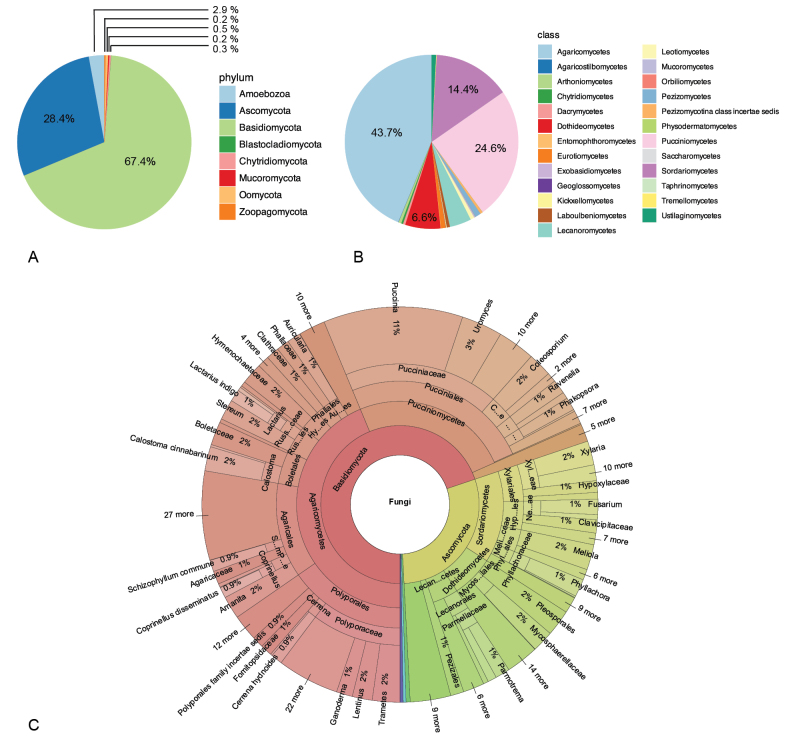
Diversity of fungi known for Honduras based on numbers of records. **A.** By phylum (fungi sensu lato); **B.** By class (fungi sensu stricto); **C.** Snapshot of the Krona plot, presenting the taxonomic distribution of records of fungi s.l.

Among the 4011 records in the fungal checklist, 96.6% refer to true fungi, 2.9% to Eumycetozoa (Amoebozoa), and 0.5% to species of Albuginaceae and Peronosporaceae (Oomycota) (Fig. 2A). Of all records of fungi sensu stricto (s.s.), i.e., without slime molds and other fungus-like organisms, 69.8% belong to Basidiomycota and 29.4% to Ascomycota. Records encompass six phyla, 25 classes, 83 orders, 236 families, 527 genera, and 1304 species of fungi s.s. The highest number of records come from the classes Agaricomycetes (43.7%), Pucciniomycetes (24.6%), Sordariomycetes (14.4%), Dothideomycetes (6.6%), and Lecanoromycetes (4.1%) (Fig. 2B). An interactive version of the Krona plot (Fig. 2C) can be accessed in the Suppl. material (see Suppl. material 3).

**Figure 3.**
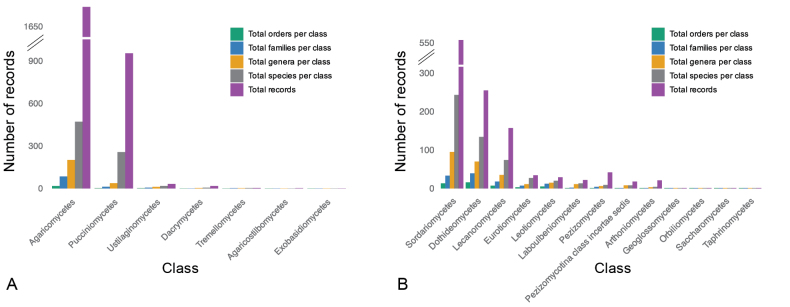
Taxonomic diversity of the records of fungi in the checklist for Honduras. **A.** Within Basidiomycota; **B.** Within Ascomycota.

Agaricomycetes represents 62.7% of all Basidiomycota records and is the most abundant class in this phylum, followed by Pucciniomycetes with 35.3% of records (Fig. 3A). Similarly, Sordariomycetes represents 48.9% of all Ascomycota records and is thus the most abundant class in the phylum, followed by Dothideomycetes with 22.4% of records (Fig. 3B). Conversely, Geoglossomycetes, Orbiliomycetes, Saccharomycetes, and Taphrinomycetes have only one record each in the checklist. Of all records of fungi s.s., 27.8% belong to two orders within Agaricomycetes: Polyporales (15.4%) and Agaricales (12.4%). These orders contain several of the typical mushroom-forming species commonly known among the public. Among the genera with the most records in the checklist is *Puccinia*, representing 11.4% of fungal records from Honduras. Taxa of macrofungi (e.g., *Amanita*, *Coprinellus*, *Mycena*) and some plant pathogens (e.g., *Cercospora*, *Phyllachora*) are relatively well studied, whereas those less well studied tend to be less conspicuous fungi (including Chytridiomycota and Oomycota) and fungi not recognized as important in applied context.

**Figure 4.**
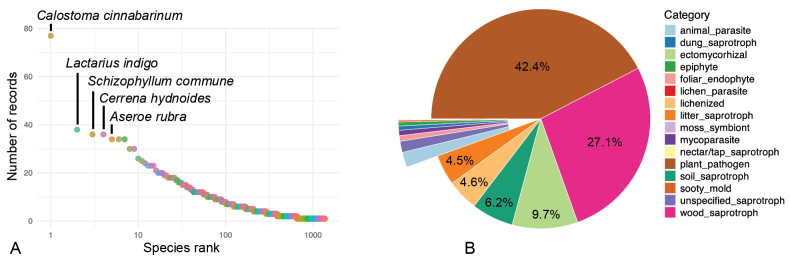
Records-based overview of the most frequently reported fungal species and ecological strategies in Honduras. **A.** Rank abundance curve showing the number of records per species. Species ranks are presented in logarithmic scale. Names of the most frequently reported species are indicated; **B.**Fungi grouped according to lifestyle based on FungalTraits data (Põlme et al. 2020).

**Figure 5.**
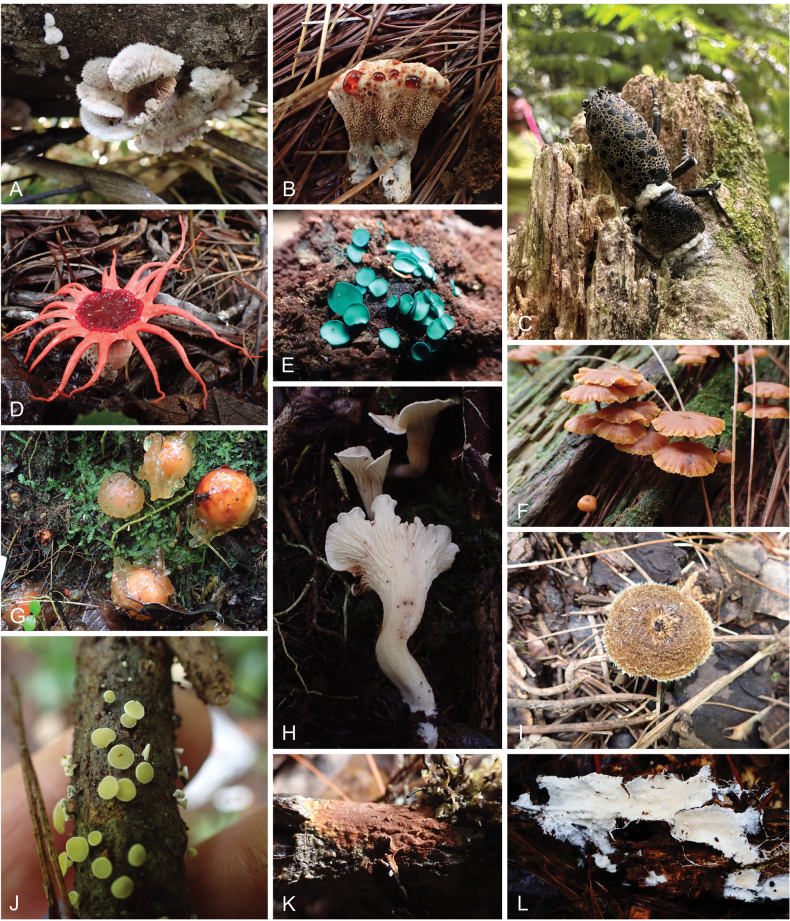
Fungi from Honduras. **A.***Schizophyllum commune*; **B.***Hydnellum* sp.; **C.***Beauveria caledonica*; **D.***Aseroe rubra*; **E.***Chlorociboria* sp.; **F.***Xeromphalina enigmatica*; **G.***Calostoma* sp.; **H.** A species of Gomphaceae; **I.***Lentinus crinitus*; **J.***Erioscyphella brasiliensis*; **K.***Hymenochaete cinnamomea*; **L.***Trechispora hondurensis*. All photos were taken in Cusuco National Park, located in northwestern Honduras.

Among the 1365 species of fungi sensu lato (s.l.) reported for Honduras, 711 species (52.1%) are reported once, 266 species (19.5%) are reported twice, and 98 species (7.2%) are reported thrice (Fig. 4A). The most frequently recorded species are *Calostoma cinnabarinum* (77 records), *Lactarius indigo* (38 records), and *Schizophyllum commune* (36 records, Fig. 5A). In addition to these three species, only *Aseroe rubra* (Fig. 5D), *Cerrena hydnoides*, *Coprinellus disseminatus*, *Coriolopsis occidentalis*, *Fabisporus sanguineus*, and *Puccinia urbaniana* are reported ≥30 times in the checklist.

For 3798 of the 4011 records in the checklist, the ecological guild is recorded in the FungalTraits database (Põlme et al. 2020) with guild assignment based on genus level. Thus, 213 records were excluded from this analysis. The majority of records, 42.4% (*n* = 1610), are classified as plant pathogens, followed by 27.1% as wood saprotrophs, 9.7% as ectomycorrhizal fungi, 6.2% as soil saprotrophs, 4.6% as lichenized fungi, and 4.5% as litter saprotrophs (Fig. 4B). Other guilds each comprise less than 1.8% of total records. In general, saprotrophic fungi represent the dominant ecological strategy, accounting for 39.6% of all fungal records in the checklist (*n* = 1504). Results based on species richness rather than numbers of records are highly similar. Plant pathogens are best represented with 545 species (43.2%), followed by wood saprotrophs with 287 species (22.7%), ectomycorrhizal fungi with 91 species (7.2%), litter saprotrophs with 79 species (6.3%), lichenized fungi with 76 species (6.0%), and soil saprotrophs with 64 species (5.1%).

## ﻿Discussion

### ﻿First checklist of fungi in Honduras

This is the first time that a comprehensive fungal checklist was compiled that catalogs all species of fungi and fungus-like organisms known from Honduras. The checklist, including records spanning 170 years (between 1852 and 2022) contains 1365 species across 4011 records. Given the 7524–8440 vascular plant species in Honduras (McCranie et al. 2018; Reyes-Chávez et al. 2021) and following the Hawksworth (1991, 2001) ratio, which estimates a 6 to 1 ratio of fungal species to vascular plant species, we expect the fungal diversity in Honduras to be in the range of 45,144 to 50,640 species. This implies that only 2.7% to 3.0% of the estimated existing fungal diversity in Honduras has been documented to date. This is not surprising, provided that only 6.2% of the estimated diversity of fungi on the planet has been described and that tropical forests remain underexplored compared to temperate regions (Piepenbring et al. 2018; Quandt and Haelewaters 2021; Niskanen et al. 2023; Kirk 2025).

The checklist remains incomplete due to the unavailability of certain publications (e.g., journals that are inaccessible with our literature search protocol). Additionally, other online databases, such as the Global Biodiversity Information Facility (https://www.gbif.org/), Cybertruffle (http://www.cybertruffle.org.uk/eng/index.htm), and the USDA Fungal Databases (https://fungi.ars.usda.gov/) may contain additional records but were not included in this version of the checklist because they are beyond the scope of this project. For future versions of the checklist, these databases and additional literature should be considered.

Records in the checklist were curated to the best of our knowledge but some errors may persist due to several factors: (1) incorrect identifications in published literature and databases; (2) potential errors in duplicate filtering (some duplicates may remain, others may have been erroneously removed, e.g., due to difficulties in verifying metadata across different data repositories); (3) manual correction of spelling errors (when a name could not be found in Index Fungorum, typographical errors in the genus name and species epithet were assessed to find the correct name); (4) potential errors in the automated classification data from the NCBI Taxonomy Database, even though inaccuracies were assessed to the best of our knowledge by comparing with Index Fungorum, MycoBank, and recent literature; and (5) dubious locality information, where it was not always clear if a locality referred to a sampling event or storage of the voucher collection. In addition, several names in the checklist refer to species complexes, e.g., *Colletotrichum gloeosporioides* (Weir et al. 2012), *Ganoderma lucidum* (Zhou et al. 2014), and *Schizophyllum commune* (Taylor et al. 2006). More detailed integrative taxonomic studies will likely reveal multiple distinct species within these complexes, requiring corrections in the checklist. Despite these potential issues, this checklist is an invaluable resource to advance mycological research in Honduras and by extension in Central America.

### ﻿Fungal diversity in Central America

Honduras is now the fifth country in Central America for which a fungal checklist has been compiled, after Panama (Piepenbring 2006, 2007; Hofmann and Piepenbring 2021), Guatemala (Flores Arzú et al. 2012), Costa Rica (Mardones et al. 2024), and Nicaragua (Soza 2025). In Costa Rica, 5534 species are documented, followed by Panama with 3103 species, Nicaragua with 1608 species, Honduras with 1365 species, and Guatemala with 350 species (Table 1). These large differences in the number of recorded species may be attributed to various factors, including the methods used to compile the checklist, collecting effort, surface area (Costa Rica 51,100 km^2^, Guatemala 108,889 km^2^, Honduras 112,492 km^2^, Nicaragua 130,373 km^2^, Panama 75,320 km^2^), habitat types, influence of deforestation and habitat destruction, and ultimately differences in fungal diversity. Note that the checklist of Guatemala focuses exclusively on macrofungi, the checklist of Costa Rica on fungi s.s., and the other three on fungi s.l.

**Table 1.** Comparison of the taxonomic distribution of fungal species in selected classes (numbers of species and percentage) in the checklists of fungi for Costa Rica (Mardones et al. 2024), Guatemala (Flores Arzú et al. 2012), Honduras (this study), Nicaragua (Soza 2025), Panama (Piepenbring 2006, 2007), and worldwide (Kirk 2025). Percentages were calculated based on the total number of fungi sensu stricto, excluding fungus-like organisms.

Class	Costa Rica		Guatemala		Honduras		Nicaragua		Panama		Worldwide	
Agaricomycetes	1269	22.9%	314	90.0%	471	36.1%	452	29.4%	524	17.8%	40,825	26.2%
Pucciniomycetes	318	5.7%	0	0%	256	19.6%	92	6.0%	144	4.9%	8,423	5.4%
Sordariomycetes	842	15.2%	10	2.9%	243	18.6%	242	15.8%	608	20.7%	25,613	16.4%
Dothideomycetes	815	14.7%	0	0%	134	10.3%	188	12.2%	427	14.5%	32,621	20.9%
Lecanoromycetes	1567	28.3%	0	0%	74	5.7%	332	21.0%	616	21.0%	12,262	7.9%
Eurotiomycetes	206	3.7%	0	0%	27	2.1%	59	3.8%	111	3.8%	4,328	2.8%
**Total**	5534		350		1304		1536		2938		155,869	

### ﻿Geographic biases

Fungal research in Honduras has been concentrated in more accessible regions, while other areas remain underexplored (Fig. 1D). This heterogeneous distribution of sampling effort across the departments of Honduras reflects similar patterns observed in other tropical countries. In Panama, for example, certain regions such as the Darién Province are inaccessible and thus poorly sampled for fungi (Piepenbring et al. 2011).

Species accumulation curves help researchers understand community structure in a given area and predict species richness provided sufficient sampling effort (Palmer 1990; Soberón and Llorente 1993; Moreno and Halffter 2001). These curves typically show a steep initial rise as common species are detected, followed by a more gradual increase as rarer taxa are encountered (Gotelli and Colwell 2001). With adequate sampling effort, curves are expected to asymptotically approach a plateau, indicating that numbers of observed species approximate the true species richness detected by the methods used (Fisher et al. 1943; McCabe 2012; Yu et al. 2022). Species accumulation curves for all departments show a steep initial slope (Fig. 1B) but only the curves for Atlántida and Francisco Morazán are beginning to level off, consistent with expectations. However, none of the curves reach a clear asymptote, preventing robust estimations of species richness. All departments require additional sampling. To date, most existing global fungal data have been collected from non-tropical countries and long-term fungal studies in tropical regions are almost nonexistent (Stallman et al. 2024a).

Due to the limited knowledge of fungal diversity across all departments, richness estimators offer limited insight into the actual species diversity present in these regions (see Suppl. material 2). The high species richness estimates for Francisco Morazán and Atlántida are likely a result of more extensive sampling compared to departments such as Colón and Gracias a Dios. Other factors that may influence species richness comprise department size (with larger areas generally supporting more species), vegetation types, and habitat heterogeneity. Other richness estimates such as the Hawksworth ratio were not analyzed on a regional scale due to a lack of reliable information regarding the vascular plant species in each department.

### ﻿Taxonomic biases

Certain phyla are absent from the checklist, including Entomophthoromycota and Glomeromycota, while others are underrepresented relative to expectations. For example, Ascomycota is represented by 536 species in the checklist, accounting for 41.1% of total fungal species reported from Honduras. This figure likely underestimates the true diversity, as Ascomycota accounts for 63.7% (99,271 described species) of all known fungal species worldwide (Kirk 2025). The relatively high representation of Basidiomycota in the checklist is likely due to conspicuous basidiomata of macrofungi – large, colorful, and easily recognized mushrooms – that facilitate detection. Conversely, other phyla such as Chytridiomycota (2 species in the checklist) and Zoopagomycota (3 species) are primarily microscopic and therefore more difficult to detect. These observations reflect a bias towards well-known macrofungi (Gonçalves et al. 2021).

The representation of classes within Basidiomycota in the checklist reflects the currently known overall species distribution: Agaricomycetes is the most species-rich class (471 species), followed by Pucciniomycetes (256 species) and Ustilaginomycetes (18 species). Some classes such as Exobasidiomycetes (1 species) and Tremellomycetes (3 species) are underrepresented in the checklist and 13 of the 20 accepted classes in Basidiomycota (sensu Wijayawardene et al. 2024) have not been reported from Honduras. Among Ascomycota, Sordariomycetes (243 species) is the most species-rich class in the checklist with 243 species (45.3%), followed by Dothideomycetes (134 species, 25.0%) and Lecanoromycetes (74 species, 13.8%). Globally, however, Dothideomycetes is the most diverse class of Ascomycota (32,621 species, 32.9%) followed by Sordariomycetes (25,613 species, 25.8%). These differences can be attributed to a collector’s bias – 294 records out of 557 total records of Sordariomycetes were sampled by two researchers: O.A. Reinking (85 records representing 30 species) and P.C. Standley (209 records representing 87 species). Only 12 of the 24 known classes in Ascomycota (sensu Wijayawardene et al. 2024) are represented in the checklist.

At the genus level, *Puccinia* (Pucciniomycetes) is the most frequently reported genus, with 441 records, the majority of which were contributed by P.C. Standley (*n* = 233) and A.S. Muller (*n* = 123). *Meliola* (Sordariomycetes) is represented by 80 records, belonging to 32 species; of these, 64 records were made by Standley. That a botanist such as Standley contributed substantially to collections of plant-pathogenic *Meliola* and *Puccinia* is not unexpected, as experienced botanists are well equipped to recognize plants exhibiting visible fungal infections. Moreover, until the early 20^th^ century, botanists routinely studied fungi to some extent, as fungi were then considered “cryptogamic plants” (Ainsworth 1981). The checklist includes 72 records of *Amanita* belonging to 20 species. Ethnomycological studies have highlighted the cultural significance of species in Amanita sect. caesarea, which are traditionally consumed and traded in indigenous communities. The “Festival Nacional del Choro y el Vino” is a gastronomic event in La Esperanza (Intibucá) that honors *Amanita* species (Mejía Chacón 2021) and coincides with the collecting season of *A. caesarea*, which is commonly known as “el choro” in this region.

The two most frequently reported species in the checklist – *Calostoma cinnabarinum* and *Lactarius indigo* – have been studied extensively for their pigments (Harmon et al. 1979; Gruber and Steglich 2007). *Schizophyllum commune* is one of the most commonly found fungi in the world, with a distribution that covers all continents except Antarctica (Ohm et al. 2010) and is used as a model organism to study mushroom development and mating type loci (Kleijburg and Wösten 2025). These top-three reported species, along with the other often reported ones (≥30 times; see Results) are all members of the mushroom-forming Agaricomycetes, except *Puccinia urbaniana* (Pucciniomycetes). In conclusion, the diversity captured in the checklist appears to be skewed by sampling effort, a well-known bias also reported elsewhere (e.g., Gonçalves et al. 2021; Lofgren and Stajich 2021; de Groot et al. 2024).

### ﻿Ecological guild biases

The overall pattern in guild distribution observed in this study is consistent with previous studies (Nguyen et al. 2016; Põlme et al. 2020). The high percentage of plant pathogens reflect a research bias towards plant-pathogenic fungi and most likely does not align with the true representation of guilds when considering the existing fungal diversity. For example, in the second half of the 20^th^ century several mycological studies in Honduras focused on fungal pathogens on banana (Goos and Timonin 1962; Goos 1963; Goos and Tschirch 1963; Stover 1963; Pinochet and Stover 1980). More recent studies refer to fungi causing plant diseases in economically important crops such as cacao and coffee (Rossman et al. 2012; Morales and Grajeda 2021; Reyes-Robles et al. 2022).

Globally, lichenized fungi represent approximately 12.7% of total fungal diversity (Kirk et al. 2008). In contrast, this group of fungi is underrepresented in the checklist for Honduras, comprising only 6.0% of reported species – likely a consequence of limited research efforts by lichenologists in the country. Arbuscular mycorrhizal fungi (AMF) are entirely absent from the checklist. This is an artifact of mycological research to date as AMF are known to dominate the root symbiosis of tropical lowland vegetation (Piepenbring 2015). Other categories present in the FungalTraits database are lacking in the checklist, such as animal endosymbionts, because they are highly unlikely to be found through macrofungal surveys. Future fungal studies should incorporate different detection strategies, including environmental DNA (eDNA) barcoding and root-associated metabarcoding to more accurately capture fungal diversity across ecological guilds.

Animal-associated fungal pathogens account for 3.2% of the fungal species, a proportion consistent with global estimates. For example, Cheek et al. (2020) reported that 3.5% of all newly described fungal species in 2019 were animal-associated (in Kickxellomycetes, Laboulbeniomycetes, Saccharomycetes, and Sordariomycetes). In Honduras, studies have focused on chytridiomycosis in amphibians from Cusuco National Park (Kolby and Padgett-Flohr 2009; Kolby et al. 2015; Thorp et al. 2021) and Pico Bonito National Park (Puschendorf et al. 2006) and arthropod-associated ectobiont and pathogenic fungi (Chaverri et al. 2008; Van Caenegem et al. 2023). However, these studies have remained limited in taxonomic, geographic, and disciplinary scope, meaning there is substantial potential for future work in this area.

### ﻿Future perspectives

Despite animal-associated fungal pathogens playing important roles in regulating host populations and shaping ecological dynamics, they have received little attention in Honduras to date. Fungal infections are a major public health concern, causing an estimated 1.6 million deaths annually (Brown et al. 2012; Almeida et al. 2019). Further, animal-pathogenic fungi, including *Batrachochytrium dendrobatidis*, *B. salamandrivorans*, and *Pseudogymnoascus destructans*, pose serious threats to wildlife, contributing to population declines and extinction risks among amphibians, reptiles, and bats (Casadevall 2017; Fitzpatrick et al. 2018). More specifically, fungal infections of arthropods contribute to declines in local communities and have the potential to impact agriculture – for example through brood infections in bees caused by *Ascosphaera* and *Aspergillus* species (Jensen et al. 2013). Studies of arthropod-associated fungi (e.g., Hypocreales, Laboulbeniales) would greatly benefit from interdisciplinary collaborations with entomologists as previously suggested (Haelewaters et al. 2021).

The taxonomic and ecological biases pointed out above, combined with the low number of species compared to Costa Rica and Panama, indicate that Honduras remains substantially undersampled in terms of fungal diversity. Notably, 71.6% of all fungal species reported from the country have been recorded only once (52.1%) or twice (19.5%), highlighting the need for expanded and long-term surveys. We recommend prioritizing regions that have received little to no attention from mycologists, particularly those with high biodiversity and conservation value as well as threatened areas. For instance, the western departments – Copán, Intibucá, Lempira, Ocotepeque, and Santa Bárbara – are home to many national parks (e.g., Celaque National Park and Montaña Santa Bárbara National Park) but remain severely understudied.

Cortés Department warrants particular attention, with its diverse ecosystems – including semi-arid pine forest, moist pine forest, moist broadleaf forest, and dwarf forest (bosque enano) – and three national parks (Cerro Azul Meámbar, Cusuco, and Omoa National Parks). Many of these national parks are considered so-called ‘paper parks’ as they face alarming rates of habitat degradation (DiBio 2017; Martin et al. 2021). This situation highlights the need for targeted research, increased funding, and intensified conservation efforts. A long-term fungal research project was launched in 2019 in Cusuco National Park and has already resulted in multiple species new to science, including *Chlorosplenium cusucoense* (Leotiomycetes), *Gloeandromyces cusucoensis* (Laboulbeniomycetes), and *Trechispora hondurensis* (Agaricomycetes, Fig. 5L) (Haelewaters et al. 2020, 2021; Van Caenegem et al. 2023; Stallman et al. 2024b), with several others in preparation in the genera *Calostoma*, *Cyathus*, *Deconica*, *Lactifluus* (Agaricomycetes), and *Chlorociboria* (Leotiomycetes). These discoveries in a short amount of time highlight the potential for further significant findings, yet broader and long-term investments in fungal biodiversity research are required across the country to reveal the existing fungal diversity of Honduras.

## ﻿Conclusion

This preliminary checklist of fungi from Honduras highlights both the richness and the current knowledge gaps in the country’s funga. Consistent with global trends, fungal diversity in Honduras remains vastly understudied, as evidenced by the majority of species being reported only once or twice and unsaturated accumulation curves. Data remain uneven across regions, time periods, ecological guilds, and taxonomic groups. We recommend more mycological studies in poorly sampled departments (e.g., Cortés), ecosystems (e.g., cloud forests), and important functional guilds (e.g., animal parasites) and taxonomic groups (e.g., Glomeromycota). Interdisciplinary approaches – integrating taxonomy, ecology, genetics, and environmental sampling – will be key to advancing fungal research. Collaboration with experts in related disciplines (e.g., entomology, pathology) can further enhance fungal discovery and ecology. This checklist offers a valuable baseline for species identification and prioritization, and serves as a step toward a more comprehensive understanding of fungal biodiversity in Honduras and the broader region.

## ﻿Additional information

### Use of AI

No use of AI was reported.

### Author ORCIDs

Libelje Mortier https://orcid.org/0009-0000-6995-7239

Lee Davies https://orcid.org/0009-0004-9361-2651

Paul M. Kirk https://orcid.org/0000-0002-0658-7338

Jose G. Maciá-Vicente https://orcid.org/0000-0002-7174-7270

Meike Piepenbring https://orcid.org/0000-0002-7043-5769

Danny Haelewaters https://orcid.org/0000-0002-6424-0834

### Data availability

Raw data are available online at https://github.com/dannyhaelewaters/haelewaters-group/tree/main/Honduras_checklist_of_fungi_2025.

## Supplementary materials

Supplementary material 1 Complete checklist of fungi for Honduras Data type xlsx https://binary.pensoft.net/file/1485580This dataset is made available under the Open Database License (http://opendatacommons.org/licenses/odbl/1.0/). The Open Database License (ODbL) is a license agreement intended to allow users to freely share, modify, and use this Dataset while maintaining this same freedom for others, provided that the original source and author(s) are credited.Libelje Mortier, Ruben Vanhoomissen, Lee Davies, Paul M. Kirk, Jose G. Maciá-Vicente, Meike Piepenbring, Danny Haelewaters

Supplementary material 2 Species richness estimates Data type pdf https://binary.pensoft.net/file/1485581This dataset is made available under the Open Database License (http://opendatacommons.org/licenses/odbl/1.0/). The Open Database License (ODbL) is a license agreement intended to allow users to freely share, modify, and use this Dataset while maintaining this same freedom for others, provided that the original source and author(s) are credited.Libelje Mortier, Ruben Vanhoomissen, Lee Davies, Paul M. Kirk, Jose G. Maciá-Vicente, Meike Piepenbring, Danny Haelewaters

Supplementary material 3 Interactive Krona plot Data type html https://binary.pensoft.net/file/1485582This dataset is made available under the Open Database License (http://opendatacommons.org/licenses/odbl/1.0/). The Open Database License (ODbL) is a license agreement intended to allow users to freely share, modify, and use this Dataset while maintaining this same freedom for others, provided that the original source and author(s) are credited.Libelje Mortier, Ruben Vanhoomissen, Lee Davies, Paul M. Kirk, Jose G. Maciá-Vicente, Meike Piepenbring, Danny Haelewaters
