# Supplementary material for: ﻿The first checklist of fungi known for Honduras: revealing taxonomic, geographical, and functional trends
Source: MycoKeys. 2025 Dec 5;126:93–117. doi: 10.3897/mycokeys.126.169230 (PMC12701356; doi:10.3897/mycokeys.126.169230)
Supplement: Supplementary material 2 — Species richness estimates [file mycokeys-126-093-s002.pdf]

A first checklist of fungi known for Honduras: revealing taxonomic, geographical, and functional trends

Libelje Mortier, Ruben Vanhoomissen, Lee Davies, Paul Kirk, Jose G. Maciá Vicente, Meike Piepenbring, Danny Haelewaters

Supplementary file 3

Methods for species richness estimates

The "specpool" function of R package *vegan* version 2.6.4 (Oksanen et al. 2022) was used to calculate the richness estimators Chao 1 (Chao 1984), Jacknife 1 (Burnham and Overton 1978), and Bootstrap (Efron 1979; Colwell and Coddington 1994).

Results for species richness estimates

Species richness estimates for each department estimate the highest species richness in Francisco Morazán and Atlántida. For both the Jackknife 1 and Bootstrap estimators, these are followed by El Paraíso, Cortés, and Comayagua. In contrast, Chao 1 places Olancho, El Paraíso, and Cortés next in species number (Table S3). A total of 586 records were excluded from this analysis, due to missing location information. Results show high standard errors caused by incomplete sampling.

Table S3. Estimated species diversity of fungi by department in Honduras. Species richness estimates shown as mean ± standard error.

Department	Number of collections	Number of species	Chao 1	Jack 1	Bootstrap
Atlántida	1034	399	633.3 ± 44.2	600.8 ± 14.2	487.1 ± 7.0
Choluteca	41	30	69.3 ± 24.1	51.5 ± 4.6	38.8 ± 1.9
Colón	10	8	16.1 ± 9.2	13.4 ± 2.2	10.3 ± 0.9
Comayagua	142	91	167.8 ± 27.8	147.6 ± 7.5	115.2 ± 3.3
Copán	56	38	148.5 ± 69.2	67.5 ± 5.4	49.5 ± 2.2
Cortés	151	103	250.0 ± 49.4	177.5 ± 8.6	133.2 ± 3.5
El Paraíso	157	112	275.0 ± 52.9	192.5 ± 8.9	144.8 ± 3.6
Francisco Morazán	1458	564	1066.1 ± 78.8	880.8 ± 17.8	697.0 ± 8.5
Gracias a Dios	12	8	13.7 + 7.0	12.6 + 2.0	10.0 + 1.0
Intibucá	26	20	47.0 ± 20.1	34.4 ± 3.7	26.0 ± 1.5
Islas de la Bahía	19	12	42.3 ± 37.5	19.6 ± 2.7	15.1 ± 1.2
La Paz	22	22	242.5 ± 96.3	43.0 ± 4.5	29.9 ± 1.5
Lempira	69	45	223.9 ± 121.3	77.5 ± 5.7	57.8 ± 2.3
Ocotepeque	11	9	20.1 ± 12.0	15.4 ± 2.4	11.7 ± 1.0

Olancho	49	34	431.7 ± 150.4	62.4 ± 5.3	44.7 ± 2.1
Santa Bárbara	40	25	104.0 ± 67.7	42.6 ± 4.1	31.9 ± 1.8
Valle	26	21	45.6 ± 17.2	36.4 ± 3.8	27.4 ± 1.5
Yoro	102	67	162.1 ± 40.0	114.5 ± 6.9	86.3 ± 2.9

References

- Burnham KP, Overton WS (1978) Estimation of the size of a closed population when capture probabilities vary among animals. Biometrika 65: 625–633. https://doi.org/10.1093/biomet/65.3.625
- Chao A (1984) Nonparametric estimation of the number of classes in a population. Scandinavian Journal of Statistics 11: 265–270.
- Colwell RK, Coddington JA (1994) Estimating terrestrial biodiversity through extrapolation. Philosophical Transactions of the Royal Society of London B 345: 101–118. https://doi.org/10.1098/rstb.1994.0091
- Efron B (1979) Bootstrap methods: another look at the jackknife. Annals of Statistics 7: 1–26. https://doi.org/10.1214/aos/1176344552
- Oksanen J, Simpson G, Blanchet F, Kindt R, Legendre P, Minchin P, O'Hara R, Solymos P, Stevens M, Szoecs E, Wagner H, Barbour M, Bedward M, Bolker B, Borcard D, Carvalho G, Chirico M, De Caceres M, Durand S, Evangelista H, FitzJohn R, Friendly M, Furneaux B, Hannigan G, Hill M, Lahti L, McGlinn D, Ouellette M, Ribeiro Cunha E, Smith T, Stier A, Ter Braak C, Weedon J (2022) vegan: community ecology package. R package version 2.6-4. https://cran.r-project.org/package=vegan

R Code

```
get_richness_for_province <- function(province_name) {

    species_counts_province <- species_counts %>%

        filter(department == province_name)

    species_matrix_province <- species_counts_province %>%

        pivot_wider(names_from = currentName,

                    values_from = collections_per_species,

                    values_fill = list(collections_per_species = 0))

    species_matrix_numeric <- species_matrix_province %>%

        select(-department, -collection_id) %>%

        mutate_all(as.numeric)

    chao_results_province <- specpool(as.data.frame(species_matrix_numeric))

    chao_results_province$department <- province_name

    return(chao_results_province)

}
```
