# Supplementary material for: ﻿The first checklist of fungi known for Honduras: revealing taxonomic, geographical, and functional trends
Source: MycoKeys. 2025 Dec 5;126:93–117. doi: 10.3897/mycokeys.126.169230 (PMC12701356; doi:10.3897/mycokeys.126.169230)
Supplement: Supplementary material 3 — Interactive Krona plot [file mycokeys-126-093-s003.html]

Javascript must be enabled to view this page.

magnitude
magnitudeUnassigned

taxonomy\_table

4011

19

19

19

6

6

2

2

4

4

13

13

7

2

5

3

2

1

3

3

3875

9

9

9

3

2

2

1

1

1

1

1

1

1

1

2

2

2

2

1

1

1

1

2705

1695

11

11

6

1

4

1

1

1

4

3

1

170

1

1

1

3

3

1

2

74

3

3

18

6

4

1

2

2

3

6

2

3

1

1

1

1

1

1

1

7

5

2

8

2

1

4

1

5

2

3

1

1

1

1

3

2

1

2

2

15

2

12

1

2

2

2

2

2

84

84

7

77

2

2

2

3

3

1

2

1

1

1

44

43

43

2

2

3

4

7

9

1

4

11

1

1

1

2

2

1

1

1

1

105

2

2

2

68

21

9

1

1

4

2

3

1

2

2

8

2

2

1

1

2

6

2

4

1

1

1

1

7

4

3

2

2

7

1

3

2

1

13

3

8

2

24

18

2

1

2

1

12

6

6

6

6

6

5

2

2

2

2

1

1

595

397

5

1

1

2

1

17

1

16

2

2

9

2

7

33

2

1

30

2

2

4

3

1

12

1

11

6

6

51

1

2

2

3

1

2

6

9

2

4

10

2

1

3

3

3

2

1

21

1

20

1

1

12

1

2

1

7

1

34

9

1

1

1

1

19

2

4

2

2

87

12

15

8

4

6

2

7

2

3

2

1

8

1

3

1

1

1

2

4

4

7

5

2

4

1

2

1

12

12

2

2

1

1

1

1

4

2

2

63

1

3

1

3

8

26

2

1

2

3

1

12

9

1

1

6

6

2

2

2

2

1

1

42

42

5

1

36

37

1

1

2

1

1

23

23

4

1

1

2

7

6

1

6

6

4

2

21

3

2

1

18

1

1

12

1

1

2

18

18

1

1

3

1

12

1

1

1

1

1

1

7

5

5

2

2

3

2

1

1

1

1

6

1

1

4

4

1

1

36

2

2

34

34

3

3

3

6

5

1

2

2

1

1

22

22

21

2

2

17

1

1

1

1

1

1

81

40

6

6

27

1

3

1

20

2

6

4

2

1

1

41

7

1

6

34

34

8

8

8

8

13

13

13

1

4

5

2

1

9

3

1

1

2

1

1

4

1

1

3

2

1

2

1

1

1

1

2

2

2

2

2

2

1

1

1

1

136

65

65

8

6

6

3

1

1

2

15

23

3

3

3

6

6

2

4

4

4

2

1

1

56

50

1

1

38

10

6

1

4

1

2

1

1

1

1

480

20

3

3

2

1

1

15

1

1

1

4

1

2

2

1

2

42

7

7

5

2

1

2

7

2

1

1

3

10

1

6

3

11

1

10

1

1

1

1

17

17

3

1

1

4

1

1

5

1

5

5

2

3

30

1

1

28

14

1

2

3

4

4

1

1

3

2

2

1

1

18

1

1

11

1

8

2

5

1

1

1

2

1

1

9

9

7

2

33

1

1

1

1

1

1

6

6

8

2

3

1

2

16

4

3

9

5

4

4

1

1

13

13

1

12

37

37

1

36

9

5

1

1

1

2

2

1

1

2

2

2

1

1

1

1

5

5

1

4

6

6

1

1

1

1

1

1

1

1

1

47

4

4

3

3

36

34

1

1

1

1

3

3

3

2

1

1

1

1

11

3

3

3

1

1

1

1

1

1

1

2

2

1

1

7

7

1

1

1

2

2

1

1

1

72

72

1

2

1

2

5

3

1

7

1

1

14

5

1

4

2

1

18

1

1

1

7

7

6

1

2

1

1

1

1

2

2

2

21

21

1

12

6

2

10

6

1

5

1

1

1

1

2

1

1

9

2

2

1

1

6

6

3

3

3

30

1

1

29

1

1

1

1

6

7

1

4

4

1

2

14

3

3

3

11

9

4

5

1

1

1

1

17

17

17

10

10

2

2

5

1

3

1

954

950

16

4

3

1

12

2

10

13

2

2

3

1

2

8

1

6

1

3

3

1

2

1

1

1

47

4

4

41

12

2

1

6

2

1

4

2

2

2

1

3

3

1

1

1

1

106

2

2

28

2

5

17

3

1

76

9

2

5

6

1

1

4

18

4

25

1

5

5

4

1

2

1

1

1

1

2

2

2

66

1

1

3

1

2

7

6

1

45

1

1

2

1

5

1

1

1

4

2

9

2

11

2

2

6

3

3

4

1

1

2

689

22

1

21

13

4

3

3

1

2

130

2

13

2

2

2

6

3

6

1

19

1

2

1

4

2

1

1

2

1

10

1

1

1

14

1

4

15

11

1

27

2

2

1

5

1

1

6

1

1

1

6

2

2

7

1

6

2

2

23

1

1

1

5

5

2

4

1

2

1

7

1

1

5

9

9

441

1

10

1

13

3

24

7

1

3

8

1

1

3

1

1

1

9

7

7

1

3

1

1

1

7

1

1

10

3

2

2

1

2

3

2

3

1

11

6

1

3

1

1

6

1

4

1

1

4

1

3

7

1

14

1

2

1

1

4

5

12

1

4

2

20

4

13

2

1

6

2

1

1

30

4

5

1

1

1

1

1

2

7

2

1

2

1

4

3

1

2

1

1

1

10

2

4

5

1

7

5

4

1

2

1

1

1

1

18

7

1

2

6

6

4

4

4

1

3

32

31

25

9

1

2

1

5

1

1

1

1

2

2

3

1

2

3

3

6

1

5

1

1

1

1

1

1

4

4

2

1

1

1

1

1

1

2

2

2

2

2

1

1

1

1

1

4

4

3

3

2

1

1

1

1

13

13

4

4

4

4

9

9

9

9

9

3

3

3

1

1

2

2

6

6

6

6

6

1138

42

42

2

2

1

1

1

1

1

38

3

3

4

4

31

5

10

16

1

1

1

21

21

21

1

1

18

18

2

1

1

1

1

1

1

1

255

1

1

1

1

10

8

1

1

7

7

2

2

2

1

1

1

1

3

3

3

1

2

1

1

1

1

4

3

3

3

1

1

1

1

1

1

1

14

14

6

6

8

2

2

1

1

1

1

8

2

2

2

3

3

3

2

1

1

1

1

1

1

1

31

17

3

3

3

1

2

1

1

10

10

5

4

4

1

1

9

9

1

1

2

4

1

1

1

1

1

17

17

2

2

15

1

2

1

3

3

1

2

2

83

2

1

1

1

1

27

27

23

4

1

1

1

2

1

1

1

1

33

15

2

1

2

2

1

1

5

1

12

1

1

10

3

3

1

1

2

1

1

2

2

2

1

1

1

1

1

1

1

1

1

2

1

1

1

1

6

1

1

1

1

2

1

1

2

1

1

2

2

2

1

1

1

2

2

2

1

1

1

1

69

1

1

1

68

25

2

1

1

1

2

1

2

1

1

1

1

1

1

1

2

3

1

1

1

5

4

1

3

1

1

1

5

2

1

1

1

1

1

1

1

6

6

1

1

1

1

16

1

3

2

5

1

1

1

1

1

1

1

1

1

1

1

1

1

10

2

1

1

1

1

6

6

6

2

2

2

1

1

1

1

1

34

2

1

1

1

1

1

1

3

2

1

1

1

1

1

1

1

29

25

12

1

1

1

1

1

1

4

1

1

11

1

1

1

1

2

2

1

1

1

2

2

1

1

1

3

1

1

2

2

22

22

16

1

1

2

2

1

1

1

1

2

2

5

1

4

1

1

1

1

1

1

1

1

6

6

1

5

29

1

1

1

1

2

1

1

1

1

1

1

23

2

1

1

1

1

1

1

1

3

3

1

2

14

1

1

4

1

2

1

1

1

8

1

7

1

1

1

1

1

1

1

1

1

2

2

2

2

1

1

1

1

18

18

18

6

6

2

2

1

1

1

1

2

2

4

4

1

1

1

1

556

184

2

2

2

126

4

4

3

1

2

3

3

21

3

1

11

2

4

1

1

5

2

1

1

1

2

2

1

1

2

2

8

2

4

1

1

76

3

5

6

5

18

3

2

1

1

4

1

1

1

12

1

1

2

4

5

4

4

1

2

1

10

2

2

4

3

1

2

2

1

1

1

1

1

1

1

4

3

3

1

1

37

12

5

1

1

4

1

6

2

4

19

5

1

2

3

4

1

2

1

2

2

2

2

144

2

1

1

1

1

11

7

1

6

2

2

2

1

1

11

2

2

2

1

1

1

1

6

4

2

42

1

1

18

7

1

2

3

4

1

1

1

6

1

2

1

1

1

9

2

1

6

6

3

1

2

1

1

65

1

1

2

1

1

1

1

49

1

8

2

1

1

1

1

1

5

2

1

2

12

2

2

5

1

1

3

1

2

1

1

1

1

1

1

2

1

1

1

1

1

1

1

1

1

1

5

2

1

1

1

1

2

1

1

2

2

2

2

1

1

1

1

4

1

1

1

1

2

1

1

2

1

1

1

1

1

1

1

1

1

1

1

1

1

1

2

2

2

2

26

23

23

15

3

3

2

3

1

1

1

1

1

1

4

1

1

1

1

1

1

2

2

2

11

2

1

1

1

1

1

1

1

8

1

1

2

2

5

2

3

63

61

2

1

1

2

1

1

56

1

1

2

4

1

1

1

2

2

1

2

1

7

1

1

5

1

5

10

1

6

1

1

2

2

2

2

2

2

2

114

114

5

2

1

1

1

1

1

18

9

9

1

1

7

2

1

3

1

80

1

1

2

5

16

1

1

1

1

3

1

3

1

1

6

2

2

1

1

3

1

1

3

1

2

2

1

4

6

4

1

1

2

2

1

1

1

1

1

157

1

1

1

1

8

8

8

1

6

1

22

1

1

1

9

9

1

1

3

4

4

4

2

2

5

5

3

2

3

1

1

2

1

1

90

74

6

1

2

1

1

1

1

1

1

1

7

4

1

2

46

1

4

1

3

4

3

2

8

1

1

3

1

1

6

1

4

1

1

2

2

8

1

7

2

2

1

1

1

1

1

5

1

1

4

3

1

10

1

1

9

9

13

9

1

1

4

4

1

1

3

2

1

1

1

1

3

3

3

21

6

3

1

2

2

2

1

1

15

7

3

1

1

2

8

7

1

2

1

1

1

1

1

1

1

1

1

1

1

1

1

1

1

1

1

116

116

116

21

14

14

1

10

2

1

1

1

1

3

3

1

1

1

3

3

1

2

26

26

8

2

4

1

1

18

1

8

9

20

20

1

1

5

1

4

1

1

9

1

5

2

1

2

1

1

2

1

1

39

28

20

1

5

4

2

1

4

1

1

1

3

2

1

4

3

1

1

1

11

2

1

1

9

1

1

3

1

3

6

6

6

6

3

1

1

1

2

2

2

1

1

1

1

1

1

1

1

1

1

1
